# Controlling Chronic Diseases and Acute Infections with Vitamin D Sufficiency

**DOI:** 10.3390/nu15163623

**Published:** 2023-08-18

**Authors:** Sunil J. Wimalawansa

**Affiliations:** Department of Medicine, CardioMetabolic & Endocrine Institute, North Brunswick, NJ 08902, USA; suniljw@hotmail.com

**Keywords:** 25(OH)D, 1,25(OH)2D, immune system, SARS-CoV-2, viral infections, vitamin D deficiency

## Abstract

Apart from developmental disabilities, the prevalence of chronic diseases increases with age especially in those with co-morbidities: vitamin D deficiency plays a major role in it. Whether vitamin D deficiency initiates and/or aggravates chronic diseases or vice versa is unclear. It adversely affects all body systems but can be eliminated using proper doses of vitamin D supplementation and/or safe daily sun exposure. Maintaining the population serum 25(OH)D concentration above 40 ng/mL (i.e., sufficiency) ensures a sound immune system, minimizing symptomatic diseases and reducing infections and the prevalence of chronic diseases. This is the most cost-effective way to keep a population healthy and reduce healthcare costs. Vitamin D facilitates physiological functions, overcoming pathologies such as chronic inflammation and oxidative stress and maintaining broader immune functions. These are vital to overcoming chronic diseases and infections. Therefore, in addition to following essential public health and nutritional guidance, maintaining vitamin D sufficiency should be an integral part of better health, preventing acute and chronic diseases and minimize their complications. Those with severe vitamin D deficiency have the highest burdens of co-morbidities and are more vulnerable to developing complications and untimely deaths. Vitamin D adequacy improves innate and adaptive immune systems. It controls excessive inflammation and oxidative stress, generates antimicrobial peptides, and neutralizes antibodies via immune cells. Consequently, vitamin D sufficiency reduces infections and associated complications and deaths. Maintaining vitamin D sufficiency reduces chronic disease burden, illnesses, hospitalizations, and all-cause mortality. Vulnerable communities, such as ethnic minorities living in temperate countries, older people, those with co-morbidities, routine night workers, and institutionalized persons, have the highest prevalence of vitamin D deficiency—they would significantly benefit from vitamin D and targeted micronutrient supplementation. At least now, health departments, authorities, and health insurance companies should start assessing, prioritizing, and encouraging this economical, non-prescription, safe micronutrient to prevent and treat acute and chronic diseases. This approach will significantly reduce morbidity, mortality, and healthcare costs and ensure healthy aging.

## 1. Introduction

Vitamin D is not a hormone but is essential for human survival. Compared with white people, darker-skinned people need longer skin exposure to sunlight. However, this is impractical for many because of the sun’s intensity, less time available for exposure, and insufficient UVB rays reaching the surface in northern latitudes (and in mornings and evenings, even in the summer). Consequently, darker-skinned people likely have a higher prevalence of hypovitaminosis, lower fertility rates, higher rates of infections and complications, a higher prevalence of chronic diseases (e.g., hypertension and cardiovascular diseases), and a shorter life span, especially during the winter [1,2]. The further north people migrated and lived, the lighter their skin pigment became as a survival mechanism. In northern latitudes, even white-skinned people cannot produce adequate quantities of vitamin D during the winter. However, in Nordic countries, it is customary for people to regularly consume fatty fish like salmon and mackerel, compensating for the lack of sunlight. This gives them essential fatty acids and fat-soluble vitamins, especially vitamin D, to stay healthy. Over the past few centuries, because of lifestyle changes (e.g., predominant indoor work), despite the lighter skin color in the absence of vitamin D supplements, people have increasingly experienced vitamin D deficiency and associated illnesses, which are intensified during winter. However, these were not particularly appreciated then. The mentioned illnesses, such as respiratory viral diseases, were cyclical, and the incidences were highest during the winter.

Significant advances have been made over the past two decades related to vitamin D, especially in the immune system and its effects on chronic diseases. Parent vitamin D (D), 25-hydroxyvitamin D [25(OH)D: calcifediol], and 1,25-dihydroxyvitamin D [1,25(OH)2D: calcitriol] all have specific roles to play in the physiological activities of vitamin D. For example, the parent vitamin D is important to reach into target cells as a precursor generating calcitriol [3]. Vitamin D and 25(OH)D are precursors for the active form, calcitriol; they are crucial for peripheral target cells to generate intracellular calcitriol for their biological and physiological activities [4]. In contrast, calcitriol has hormonal and non-hormonal effects. The genomic effects occur following calcitriol binding with its receptor, vitamin D (calcitriol)receptors (VDR), migrating to the nucleus, and modulating genes [4]. In addition, calcitriol has essential non-genomic functions, including its effects on membranes [5].

## 2. Vitamin D—Brief History

Vitamin D (calciferol) is a fat-soluble secosteroid, the clinical importance of which was understood in 1920. Then, patients with tuberculosis were cared for in solariums or with daily exposure to direct sunlight [1]. They were provided a diet containing egg yolks, cod liver oil, and fatty fish that sped up their recovery and reduced mortality. In the 1930s, the chemical structure of vitamin D was established. It was understood that exposure to ultraviolet B (UVB) of sunlight generates vitamin D, which leads to the recovery of patients with tuberculosis. Based on this, some physicians speculated about a connection between sun exposure and enhancing immune functions. However, until recently, the vital association of sun exposure (that is associated with vitamin D generation) with immune system activities was not understood.

**Figure 1:**
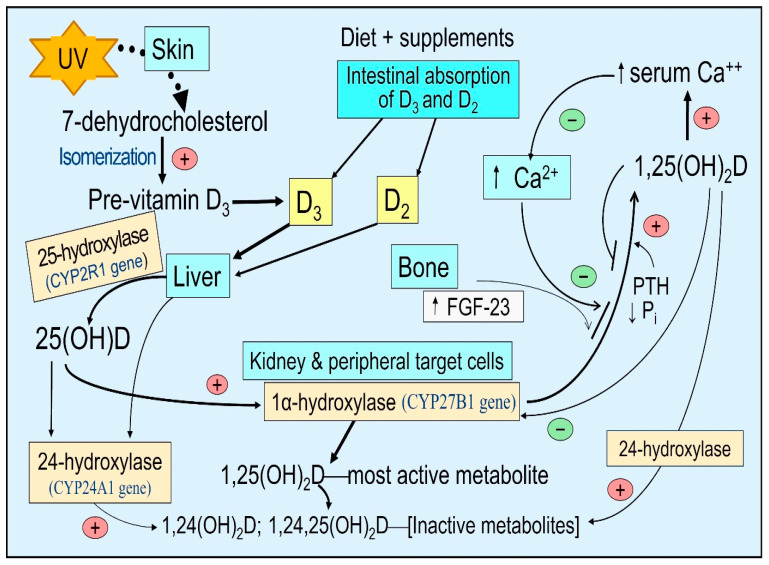
Basic steps involved in generating and catabolizing vitamin D and 25- and 1α-hydroxylase activation steps. Synthesis of D_3_ in the skin—activation of vitamin D and 25(OH)D in liver and peripheral target cells by respective cytochrome, P450-hydroxylase enzymes, and 24-hydroxylase enzyme adds an OH group at 24th position of the steroid molecule, which inactivates all vitamin D products as illustrated.

UVB converts 7-dehydrocholesterol in the skin into pre-vitamin D, which is hydroxylated to form 25(OH)D in the liver, the precursor of 1,25,(OH)_2_D (calcitriol), which helps patients’ recovery [6,7]. The skin type (thickness of the skin) and the degree of pigmentation are mainly determined genetically [8], but modifications occur from occupations and environmental exposure. The melanin pigment protects the skin from sunburn and DNA damage. However, it reduces the penetration of the skin by UVB wavelengths between 290 and 315 nm of sun rays in [9,10]. Therefore, those with darker skin (Fitzpatrick skin type V (brown) and VI (dark); https://www.ncbi.nlm.nih.gov/books/NBK481857/table/chapter6.t1/: Accessed, 5 July 2023) restrict the penetration of UV rays to the dermis [10,11]; thus, they have a reduced capacity to generate vitamin D following exposure to sunlight. Therefore, they need prolonged sunlight exposure to generate identical amounts of vitamin D. Figure 1 illustrates the steps involved in generating and catabolizing vitamin D, and 25- and 1α-hydroxylase activation steps.

Evolutionarily, having a varying degree of melanin pigment in the skin was a healthful compromise for those living in regions closer to the equator (e.g., central Africa), where the sun’s rays are intense. However, when humans migrated from central Africa to the northern areas to access more food, they had lesser exposure to sunlight. To overcome this natural disadvantage, the density of melanin pigment in the skin gradually reduced over many generations—a natural evolutionary survival mechanism. Those who developed a lighter skin color (white-skinned people) had a significant survival advantage—better protection against infections, fewer chronic diseases [12], and a higher rate of procreation and longevity [13].

The pre-vitamin D generated in the dermis undergoes thermal isomerization to form vitamin D_3_, binds to vitamin D binding protein (VDBP), and releases it to circulation within 24 h [14]. Because 25-hydroxylation in hepatocytes is a rate-limiting step, on average, irrespective of the amount of vitamin D reaching the liver, it takes about three days to raise the serum 25(OH)D concentration [15]. Circulatory concentrations of vitamin D and 25(OH)D concentrations depend on the duration of sun exposure, the intensity of skin melanin content, and the ability of the skin to generate pre-vitamin D [10]. In the case of oral vitamin D, the serum 25(OH)D concentrations depend on doses and frequency of administration [16].

The internalization rate of vitamin D into cells is higher than that of 25(OH)D in cells that do not express the megalin–cubilin transport system [17], such as the kidney and parathyroid gland. These cells can not only express the CYP27B1 gene (1α-hydroxylase enzyme) but also the CYP2R1 gene (25-hydroxylase enzyme) so that cells can efficiently generate calcifediol and calcitriol [18,19]. Most other cell types depend on a concentration-dependent gradient diffusion of D and 25(OH)D from the blood into them for their genomic actions and autocrine and paracrine signaling mechanisms. Only free components (not bound to VDBP) diffused through cell membranes. The entry (kinetics) is more restricted with 25(OH)D, as it is more tightly bound to the VDBP than D [20,21,22,23].

## 3. Generation/Obtaining Vitamin D and Transportation in Humans

Vitamin D is a secosteroid molecule: fully activated vitamin D, 1,25(OH)_2_D has broad physiological functions [7,24]. These include immune modulation with anti-inflammatory and antioxidant actions [13], membrane stability [25], metabolic and mitochondrial respiratory functions [13], and reproductive biology. Genomic functions include the favorable transcription of over 1200 genes [26,27].

Vitamin D_3_ is supposed to be obtained naturally by humans following exposure to UVB rays from sunlight. In the skin, 7-dehydrocholesterol converts to pre-vitamin D_3_, which isomerizes to form vitamin D_3_. It binds to VDBP and diffuses via capillaries into the circulation [28]. There is little vitamin D in food: e.g., D_2_ in sun-exposed mushrooms and D_3_ n fatty fish. After intestinal absorption as chylomicron, vitamin D is incorporated into VDBP via lipoproteins and reaches the bloodstream through the thoracic duct [29]. These reach hepatocytes, where 25(OH)D is generated and released into the circulation, mostly bound to VDBP [30]. In addition to hepatocytes, 25-hydroxylase (CYP2R1 gene) is present in other target cells/tissues but in minor concentrations [31,32,33] so that D can be converted to 25(OH)D in these cells, as in immune cells [34,35]. However, there is no evidence that peripheral target tissue cell-generated calcifediol contributes to circulatory 25(OH)D.

Vitamin D, 25(OH)D, and 1,25(OH)_2_D have different dissociation constants in binding to VDBP, which determine the free (unbound to VDBP) D and 25(OH)D concentration in the circulation, which is approximately 1% of the that of the total vitamin D. The dissociation constant of 25(OH)D is about 10^−9^ m, while for vitamin D and 1,25(OH)_2_D, it is approximately 10^−7^ m [20,23]. Consequently, the circulating half-lives of these three compounds are inversely associated with the dissociation constants. For 25(OH)D, its half-live is between two to three weeks (as it is tightly bound to VDBP), depending on the vitamin D status in the body, while for vitamin D, it is one day, and for 1,25(OH)_2_D, a few hours [36]. Accordingly, the free circulating proportions are highest for 1,25(OH)_2_D, then D, and lowest for 25(OH)D.

A few thousand International Units (IU) of vitamin D_3_ could be synthesized in the skin after exposure to sunlight, which takes about 24 h to materialize in the circulation [14]. Ingested vitamin D_3_ appears in circulation between 12 and 20 h after intestinal absorption and transportation [14,29,37]. The circulating half-life of D_2_ and D_3_ is approximately 24 h; that of D_2_ is less than that of D_3_ [29]. Because of this short half-life, even higher bolus doses of vitamin D are eliminated from the body in a few days [14,38]. Therefore, the best way to maintain a steady state of vitamin D and 25(OH)D in circulation is through regular daily sun exposure and/or daily supplementation [16].

## 4. Consequences of Vitamin D Deficiency

**Figure 2:**
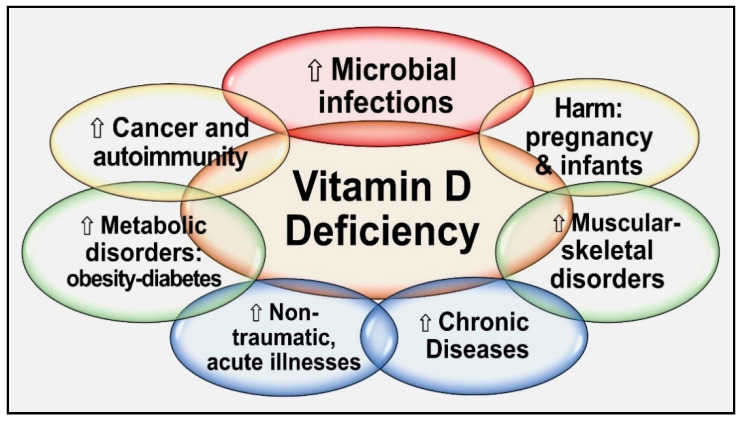
Summary of major adverse effects of vitamin D deficiency.

Vitamin D deficiency universally impairs its intended benefits in all body systems. Its deficiency increases the vulnerability to infections, increases generalized inflammation, increases risks for diseases and infections, and worsens chronic diseases [12,39,40]. Consequently, hypovitaminosis increases the susceptibility to infections and diseases and enhances the severity of illnesses [41], leading to increased complications and premature deaths [42,43,44]. Vitamin D has pleiotropic effects on body systems, especially the immune, musculoskeletal, cardiovascular, pulmonary, neurological, gastrointestinal, and renal systems. Figure 2 illustrates the expected consequences of chronic vitamin D deficiency.

Persons with chronic kidney disease (CKD) have insufficient handling of vitamin D, 25(OH)D, and 1,25(OH)_2_D. This is due to gastrointestinal malabsorption, increased catabolism, and a significant decrease in renal 1α-hydroxylation by CYP27B1. This results in low circulatory calcitriol that causes hyperphosphatemia and elevated fibroblast growth factor-23 (FGF-23) concentrations [45]; these initiate the CKD of mineral and bone disorder (CKD-MBD) [46,47]. The treatment modality of CKD-MBD has shifted from single biomarkers (measurement of calcitriol) to serial (economical) measurements of calcium, phosphate, and parathyroid hormone (PTH); these provide a broader insight and better control, helping the management of persons with CKD [46].

The abovementioned abnormalities of vitamin D metabolism lead to secondary hyperparathyroidism, which rapidly responds to oral cholecalciferol (D_3_) [48]. Survival is increased for those with all types of CKD when calcitriol is administered with vitamin D [49]. In contrast, the activation of CYP24A1 catabolizes vitamin D and its active metabolites, increasing serum 24-hydroxyvitamin D, 24,25-dihydroxyvitamin D, and 1,24,25-trihydroxyvitamin D, to the 25(OH)D ratio in the circulation (Figure 1)—known as vitamin D catabolic (metabolic) ratio [50].

The higher catabolic ratios and thus lower 25(OH)D concentrations are associated with modestly increased all-cause mortality [50]. Circulatory concentrations of D_3_ and 25(OH)D_3_ are in the micromolar range, while 1,25(OH)_2_D_3_ is present in the nanomolar range, with a calcitriol concentration of approximately nine-hundred-fold lower {Wimalawansa, 2023 #17062}. Therefore, calculating this ratio does not include calcitriol or 1,24,25-trihydroxyvitamin D concentrations in circulation as they are minuscule (as other uncommon metabolites and epimers of vitamin D), making it easier to calculate. While controversial, a reverse J shape of all-cause mortality has been reported with total serum 25-hydroxyvitamin D concentration [51], for which explanations and counters are numerous [52,53].

## 5. Muscular–Skeletal Benefits of Vitamin D

The classical actions of vitamin D involve mineral metabolism—calcium absorption and mineral conservation, skeletal calcification, and musculoskeletal functions [6,7]. These skeletal functions—bone formation/resorption and mineralization—depend on the parathyroid hormone in conjunction with the hormonal form of calcitriol derived from proximal renal tubular cells [13].

The tissue transport mechanism for steroids—megalin–cubilin endocytotic system [17]—is essential for delivering vitamin D and 25(OH)D into proximal renal tubular cells for generating calcitriol [17]. This mechanism is also present in parathyroid cells. This active transportation system is also present in fat and muscle cells—the storage tissues. The musculoskeletal system and parathyroid hormone (PTH)-driven vitamin D activities, like calcium homeostasis, are considered a part of the endocrine functions of vitamin D [54]. In contrast, the intracrine/autocrine and paracrine functions of calcitriol in peripheral target cells, like immune cells, are driven by both genomic and other signaling mechanisms. The generation of calcitriol by 1α-hydroxylase (CYP27B1) within immune cells is dependent on the ability to diffuse enough vitamin D and/or 25(OH)D from the circulation into immune cells [55,56]. This is crucial for all immune cell activities.

## 6. Hypovitaminosis D and Viral Respiratory Infections

Respiratory tract illnesses, including colds, influenza, and COVID-19, escalate in the winter. There are specific reasons why countries located far north of the equator in the northern (and southern) hemispheres experience winter-associated viral respiratory cycle that increases in colder months with less sunlight [57,58,59]. During the winter, the sunlight does not carry adequate UVB rays. In addition, rays come at a narrow-angle that does not sufficiently penetrate the skin for humans to generate vitamin D. One consequence of insufficient UVB rays is a marked reduction in circulating D and 25(OH)D concentrations. This weakens the immune system. In addition, viruses live longer outside human bodies in cold and dryer climatic conditions, such as in winter time [60,61,62].

Vitamin D deficiency markedly impairs overall immunity and thus increases the risk of illness, including metabolic disorders and infections. This makes individuals vulnerable to microbial infections [63,64], primarily viral respiratory diseases [65,66,67,68,69,70], including coronaviruses [71,72,73]. Vitamin D adequacy—having blood levels greater than 30 ng/mL (older definition) [74] but preferably greater than 50 ng/mL during winter and viral epidemics—significantly reduces the risk of respiratory viral infections [1,67,75].

Children rely primarily on their innate immune systems to counteract pathogenic microbial invasions. Since they have better innate immunity than the elderly, they are less likely to develop symptomatic COVID-19, complications or die from it unless they have severe hypovitaminosis D [76]. Severe vitamin D deficiency (i.e., serum 25(OH)D concentrations of less than 12 ng/mL) increases the risks of developing fatal immunological disorders, like Kawasaki-like disease and multi-system inflammatory syndrome [77,78]. When children with severe vitamin D deficiency are exposed to a high viral load, they could experience severe hyperimmune reactions with the complications mentioned above [77,79,80].

## 7. Extra-Skeletal Benefits of Vitamin D

Most extra-musculoskeletal biological activities of calcitriol occur following the generation of calcitriol within peripheral target cells (i.e., not via the circulatory, hormonal form), where it acts as a signaling molecule and a local cytokine. The latter functions include controlling cell proliferation and maturity, preventing cancer cell growth, brain development, respiratory and reproductive functions, and mitochondrial energy generation [24,81,82,83]. However, calcitriol’s most prominent and life-saving extra-skeletal role is modulating the immune system [84,85]. Vitamin D maintains a robust immune system, which helps to overcome infections, including COVID-19 [55,86,87], and prevents autoimmunity [88,89].

A large data set and emerging data support multiple physiological functions of vitamin D, via calcitriol. These data suggest vitamin D should be used as a preventative and adjunct therapy in several common disorders, including sepsis and COVID-19 infection [67,70,90,91]. Nevertheless, vitamin D is rarely included in clinical protocols or guidelines, or advised by leading health authorities or by governments to their fellow citizens to keep them healthy [24]. In addition, recommendations from medical and scientific societies are confusing, contradictory, and out of date [41,92]. However, public awareness of vitamin D and its beneficial effects on the immune system has improved since the COVID-19 pandemic. This is primarily due to relentless positive work by small groups of scientists, despite the negative publicity by big pharmaceutical corporations. Examples include the clinical guidelines from the Front-Line COVID-19 Critical Care Alliance (https://COVID19criticalcare.com/treatment-protocols/: Accessed 5 July 2023), affirmative Substack articles, and websites like https://COVID19criticalcare.com (Accessed 1 July 2023) [93].

Sufficient calcitriol synthesis within immune cells prevents autoimmune reactions profoundly and controls inflammation and infections [39,40]. These physiological actions manifest by suppressing the expression of inflammatory cytokines and increasing the expression of anti-inflammatory cytokines and anti-oxidative compounds [70,94]. Most chronic diseases are associated with chronic inflammation that maintains the disease process [39]. In addition, calcitriol enhances the production and release of antimicrobial peptides, cathelicidin, and beta-defensin via its autocrine and paracrine actions (Figure 1).

**Figure 3:**
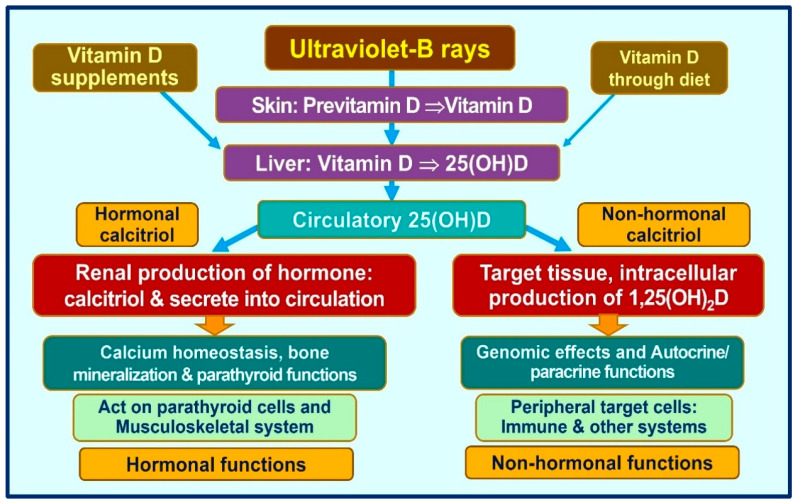
Humans should predominantly generate vitamin D via exposure to ultraviolet-B rays. Vitamin D is also obtained in via diet supplements, but the quantities are small. Figure exemplifies the main differences between the circulatory hormonal form of calcitriol (generated via renal tubular cells) vs. the intracellularly generated calcitriol in peripheral target cells (like all immune cells).

These antimicrobial peptides stimulate white blood cells, macrophages, and natural killer cells and direct the circulating viruses to macrophages to destroy them [95]. Vitamin D signaling plays a crucial role in intrinsic defense against intracellular microorganisms via generating antimicrobial proteins like cathelicidin [40]. In addition to directly binding to and killing a range of pathogens, cathelicidin acts as a secondary messenger, augmenting vitamin D-mediated reduction in inflammation during infection [96]. In addition, calcitriol stabilizes tight junctions of epithelial cells of the respiratory tract and cardiovascular system, protecting them from fluid leakage and viral dissemination into soft tissues [97,98]. Figure 3 illustrates the generation of calcitriol and the critical difference between the hormonal form and the non-hormonal form of calcitriol.

## 8. Importance of Circulatory Vitamin D and 25(OH)D for Target Cell Generation of Calcitriol

Over the years, the focus has been on cholecalciferol (D_3_) to prevent musculoskeletal disorders [99]. However, in the past two decades, several fundamental advances have been made by researchers in understanding the biology and physiology of calcifediol and calcitriol and delineating how and when to use them as therapies. Over the past decade, emerging evidence has added more value and highlighted the importance of these vitamin D compounds in human biology and clinical immunology [17]. While the musculoskeletal system functions could be maintained with smaller doses, of between 800 and 2000 IU/day, higher amounts, like 5000 to 10,000 IU per day or 50,000 IU once a week, are necessary for a non-obese 70 kg adult to maintain serum 25(OH)D concentrations above 50 ng/mL, which are needed to overcome infections [55,56].

Those who are obese, taking medications that increase catabolic activity of vitamin D (e.g., anti-epileptic and retroviral agents), or have significant fat malabsorption require several fold-higher doses than those mentioned above. Even with such amounts, a vitamin D-deficient person likely takes several months to increase their serum 25(OH)D to therapeutic levels of over 50 ng/mL [56]. Using the mentioned doses of vitamin D, even in a vitamin D-sufficient person (guidelines for community-dwelling persons) to reach and maintain a serum 25(OH)D concentration of above 40 ng/mL would take a few weeks to raise the serum 25(OH)D concentration above 50 ng/mL [55]. Therefore, such doses could be insufficient (and ineffective) to achieve the desired target serum 25(OH)D concentration in emergencies.

Serum 25(OH)D concentrations are reduced in chronic diseases like metabolic disorders, obesity, cancer, infections, and all-cause mortality [100,101,102,103]. Notably, less frequent administration (i.e., intervals of less than once a month—(i.e., intermittent bolus dosing) and even higher doses, like 300,000 once in six months, do not generate the intended clinical outcomes. This is because the half-life of vitamin D is about one day, and that of 25(OH)D is between two to three weeks (depending on the vitamin D status). No matter how high the doses is, the serum 25(OH)D concentration would not be sufficiently high for more than three months [104,105,106]. In addition, infrequent administrations lead to unphysiological fluctuation of serum and tissue levels of vitamin D metabolites (see below).

## 9. Clinical Study Outcomes Using Higher Doses of Vitamin D

Meta-analyses of RCTs concerning vitamin D supplementation reported a significant reduction in the incidence and severity of respiratory tract infections [107,108,109]—better clinical outcomes were reported with daily vitamin D than with infrequent administration. In contrast, when vitamin D is administered at longer intervals than once a month, fewer benefits are observed, and the outcomes are not satisfactory [110,111].

Using higher doses of vitamin D consistently has been reported to have better clinical outcomes than the government-recommended doses of 800 IU/day, which have no tangible effect on any disease other than muscular–skeletal disorders [107,112]. For example, adequate supplementation with vitamin D reduces cancer [113], leads to the regression of prostate cancer [114], lowers blood pressure (especially in African Americans) [115], and reduces insulin resistance [116,117], including in obese children [118], and prevents multiple sclerosis [108,119].

However, studies that used minute doses of vitamin D based on outdated recommendations (i.e., using 280 IU/day or less than 1000 IU/day) [109,120], as with the Women’s Health Initiative study of cancer prevention and infrequent administration of 100,000 IU vitamin D_3_ quarterly [121] failed to prevent cancer and other disorders. Based on vitamin D biology and physiology, this is not surprising. Most clinical studies reported an inverse association between vitamin D status and mortality [103,122], and the relation is curvilinear [41].

## 10. Entry of D and 25(OH)D into Peripheral Target Cells

Most steroid hormones enter cells via diffusion and endocytosis via the membrane-based megalin–cubilin system as in the kidney and parathyroid gland, muscle, and fat cells [17]. In addition, this mechanism of active cellular entry is essential for generating the hormonal form of calcitriol in renal tubules and parathyroid glands—for vitamin D’s endocrine functions [17,54]. However, unlike the cells mentioned above, other peripheral target cells, like immune cells, do not have an active vitamin D megalin–cubilin transportation system [56]. Thus, in addition to some endocytosis, these cells mainly depend on a concentration-dependent gradient for diffusions of vitamin D and 25(OH)D (mostly bound to VDBP) into them [123].

**Figure 4:**
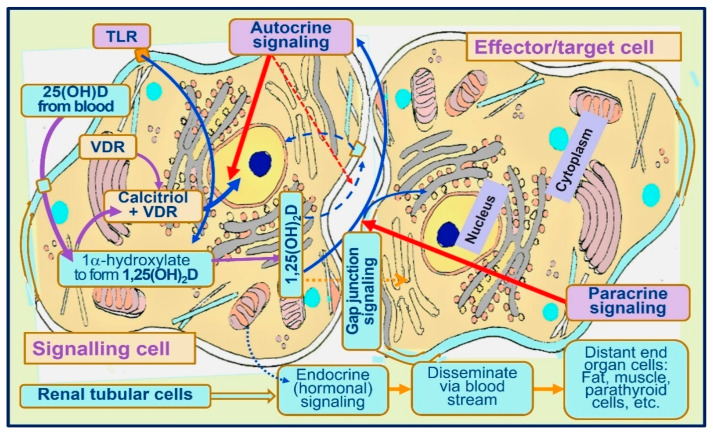
Pathways and mechanisms of actions of calcitriol activating immune cell functions: Activation of D and 25(OH)D into calcitriol [1,25(OH)_2_D] intracellularly leads to genomic actions, autocrine (activation of functions within the same cells) and paracrine (indicating cell to effector cells) signaling.

In addition to diffusion, as illustrated above, VDBP bound D and 25(OH)D enters these cells via endocytosis [22]. Since the affinity of vitamin D to VDBP is less than 25(OH)D, given the same concentration in the blood, more vitamin D could enter immune cells. However, since the half-life of vitamin D is only one day, the total entry of vitamin D is less than 25(OH)D. Figure 4 illustrates the mode of access of vitamin D and 25(OH)D into peripheral target cells, like immune cells [55], from the circulation that leads to the generation of intracellular calcitriol [41], which is crucial not only for the genomic functions but also autocrine and paracrine functions of immune cells and other target cells [87,124,125,126].

When vitamin D is taken daily, the circulatory vitamin D concentrations are likely to be higher than 25(OH)D concentrations [41]. Therefore, more vitamin D could diffuse into peripheral target cells than 25(OH)D because of the higher concentration gradient of D. When this happens, more vitamin D than 25(OH)D would reach into target cells and hydroxylated to form calcitriol. If this is the case, the measurement of serum 25(OH)D alone, as carried out in routine clinical practice, may not provide the correct information about vitamin D adequacy or the replacement requirements for physiological functions, including a robust immune system (Figure 4). The opposite happens when the same dose of vitamin is consumed once a week; a higher concentration of 25(OH)D is present in the circulation than in vitamin D.

## 11. Vitamin D, Epithelial Barriers, and Gap Junction Stability

D_3_ enhances epithelial and endothelial stability independently of canonical pathways through calcitriol/CTR-derived genomic outcome [127]. The disruption of endothelial stability and vascular leak enhancement are prevented via D_3_ supplementation. These rapid membrane-related actions of vitamin D are derived from D_3_ and its two common metabolites, 25(OH)D and 1,25(OH)_2_D, at a similar potency.

The deficiency of D_3_ and its metabolites impairs endothelial barriers, leading to vascular fluid leakage into soft tissues [127]. Similarly, weakening gap junctions and epithelial barriers lead to viral infiltration and the propagation of infections, as seen in sepsis and viral infections like SARS-CoV-2 [128]. These non-transcriptional mechanisms are also essential in controlling inflammation and preventing endothelial and epithelial cell destabilization.

## 12. What Has Changed over the Years Related to Vitamin D?

A century ago, it was established that sunrays (vitamin D) reverse rickets in children and are effective against tuberculosis. In addition, a large body of scientific evidence demonstrates that vitamin D plays a central role in disease prevention (maintaining a disease-free state) and preventing severe symptoms, diseases, complications, and deaths [44]. A few years ago, exposure to sufficient UVB rays was believed to generate no more than 3000 IU/day. However, recent data confirmed a person with a lighter skin color could generate a few thousand IUs of vitamin D_3_ after one hour of UVB exposure over a third of the upper body [129,130,131].

Maintaining a steady state of D and 25(OH)D in circulation is helpful for better physiological functions. Marked fluctuating serum 25(OHD concentration due to prolonged interval administration is unphysiological and likely to over-activate 24-hydroxylase enzyme, CYP24A1 concentrations, increasing the catabolism of active metabolites of vitamin D. Based on half-lives in circulation, the frequency of administration of vitamin D must not exceed once in ten days (or once a week), and no more than once a month. Therefore, vitamin D should not be administered at intervals longer than two-week intervals [132]. This will allow for keeping a steady circulatory concentration [110,133].

**Figure 5:**
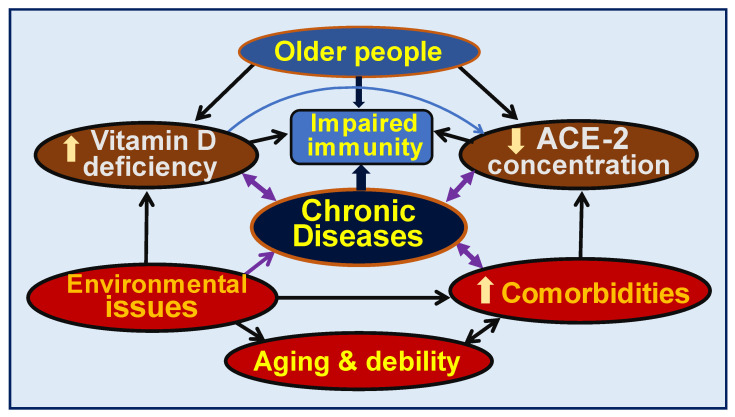
Schematic representation of how chronic diseases increase morbidity and mortality in older people. These are exacerbated by hypovitaminosis D, low angiotensin converting enzyme-2 (ACE-2) concentrations, environmental issues/pollution, and co-morbidities.

The importance of the above is highlighted by six positive respiratory tract infection-related RCTs, most conducted in children [42,43,57,58,59]; all of these used daily doses of vitamin D [101,102,134]. Another meta-analysis of RCTs on vitamin D supplementation in respiratory tract infections reported that vitamin D is most effective as a treatment when administered in daily doses than intermittently [135]. Chronic diseases are most common among older people partly due to longer-term vitamin D deficiency [136], and are associated with an increased rate of deaths [44,122]. They also have multiple co-morbidities associated with hypovitaminosis D and low-circulating ACE-2 receptors, increasing the vulnerability to infections and other pathological ailments (Figure 5).

## 13. Vitamin D Intake Should Depend on Body Weight and Target Serum 25(OH)D Concentration

Different dosing schedules have varied effects on serum vitamin D and 25(OH)D concentrations—daily doses maintain a stable circulating concentration [16]. In contrast, ingesting vitamin D for longer than monthly intervals results in significant circulatory 25(OH)D concentration fluctuations; this is not physiological and may not benefit much [110,132,133]. Schedules used for vitamin D supplementation as prophylactic and treatment or in RCTs will profoundly affect the serum D and 25(OH)D concentrations (primarily due to the short half-life of vitamin D); thus, this needs to be considered for better clinical outcomes.

Vitamin D supplementation and sufficient UV exposure increase maternal circulating 25(OH)D concentration in breast milk [137,138]. It has been known that solely breastfed infants exhibit vitamin D deficiency [139], which is easily correctible with vitamin D drops given to nursing infants [140]. For each 1000 IU/d of vitamin D_3_-supplemented to a lactating mother, vitamin D concentration in her breast milk increased by about 80 IU/L. The recommended average dose of vitamin D_3_ for pregnant and lactating mothers is 6000 IU/d; this provides the infants with 400–500 IU of vitamin D per day [120].

The circulating concentration of 25(OH)D in the fetus is approximately 70% of that of the mother; thus, a diffusion of 25(OH)D occurs across the placenta [141]. However, since vitamin D concentrations are slightly below the maternal concentrations, relatively lower amounts are diffused via the placenta [16]. The same phenomenon has been reported in transferring vitamin D and 25(OH)D to breast milk [141,142]. The diffusion gradient can be increased by raising the maternal serum 25(OH)D to 50 ng/mL [143].

## 14. Vitamin D Is Essential for Activating Immune Cells

Calcitriol is the most active vitamin D metabolite, crucial for combating invading pathogens and preventing autoimmunity, and chronic diseases [1,2]. Through multiple mechanisms, calcitriol modulates the immune system. When secreted into the bloodstream from renal tubular cells, calcitriol functions as a hormone. This alters the behavior of cells involved in calcium-phosphate-bone metabolism, intestinal, bone and parathyroid cells. The average circulatory concentration of calcitriol in the circulation about 0.045 ng/mL, but the concentration of its free, the diffusible form is even less. It is far below the threshold needed to initiate intracellular signaling. Consequently, such the pmolar concentration of hormonal calcitriol, is over 20 times less, thus have no tangible effect on intracellular intracrine signal transduction or genomic functions in immune cells. Besides, vitamin D and 25(OH)D concentrations in the circulation are about 900-fold higher than circulating vitamin D and 25(OH)D concentrations. Consequently, circulating calcitriol has no evident impact outside the muscular-skeletal and fat cells.

Peripheral target cells, like immune cells, depend on vitamin D and 25(OH)D primarily via diffusion from the circulation to generate higher concentrations of non-hormonal calcitriol. When immune cells detect external threats, like circulating microbes or unfamiliar antigens by pattern recognition receptors, like membrane-bound Toll-like receptors TLR (TLR-4) they send signals to increase the expression of 1α of VDR, thus, increasing in the cytoplasm.

Higher nmol range concentrations of calcitriol generated in-tracellularly in response to TLR signaling, provides (hysiological) intracellular autocrine/intracrine signaling that is crucial for immune functions to overcome threats, like infections. Consequently, there is a hold mechanism, increasing serum, i.e., beyond threat like detecting unfamiliar proteins or antigens in the circulation or local tissues. The sporadic increases in the synthesis of calcitriol and VDR in responses to TLR-4 signaling, ensures the formation of sufficient calcitriol-VDR complexes to modulate transcriptions, and intra-cellular autocrine signaling and genomic modulation, as when needed.

**Figure 6:**
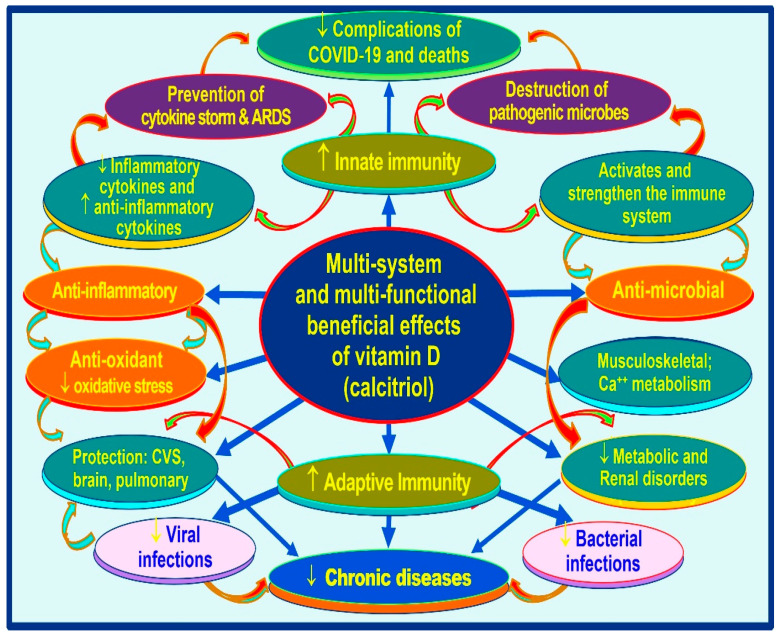
A schematic summary of multi-system beneficial effects of maintaining sufficient vitamin D and 25(OH)D in the circulation. In contrast, chronic vitamin D deficiency causes dysfunction of the immune system, increases the risk for infections and their complications, enhances the vulnerability to and severity of conditions, and increases the prevalence of chronic diseases (according to Wimalawasna, 2020: [146]).

Regulates inflammation and oxidative stresses through the abovementioned mechanisms, primarily by suppressing inflammatory cytokines and enhancing the synthesis of anti-inflammatory cytokines. Immunomodulatory effects of vitamin D include activation of immune cells such as T and B cells, macrophage and dendritic cells, and enhanced production of several antimicrobial peptides and neutralizing antibodies [3,4,5]. Figure 6 illustrates broader vitamin D (calcitriol) actions in innate and adaptive immune systems.

Once adequate concentrations are generated within the immune cells, calcitriol activates and bonds to the cytosol’s vitamin D (calcitriol)receptors (VDRs) that translocate into the nucleus for its genomic actions. The interaction of calcitriol with its receptor leads to the translocation of the complex to the nucleus, where it binds to the genome and modulates over 1200 genes [144]. In addition, intracellular calcitriol acts as an autocrine and paracrine signaling. Calcitriol down-regulates inflammation and oxidative stress through multiple mechanisms, primarily by suppressing inflammatory cytokines. Immunomodulatory effects of vitamin D include the activation of immune cells such as T and B cells, macrophage and dendritic cells, and enhanced production of several antimicrobial peptides and neutralizing antibodies [87,126,145]. Figure 6 illustrates broader vitamin D (calcitriol) actions in innate and adaptive immune systems.

Because hypovitaminosis D status does not activate immune cells, it causes relative immune paresis and delayed responses. This increases people’s vulnerability, especially to bacteria (like tuberculosis) [13] and respiratory viruses [147,148], including COVID-19 [149,150]. Recent clinical studies have supported the latter [76]. For example, serum 25(OH)D concentrations are significantly lower in those who are PCR-positive for SARS-CoV-2 (mean concentration of 11.1 ng/mL; *p* = 0.004) compared with those with negative results (24.6 ng/mL), demonstrating a higher vulnerability [151]. This is striking when using the pre-infection serum 25(OH)D concentration to correlate with infection vulnerability [152].

In addition, there is a strong correlation between severe vitamin D deficiency and cytokine storm—a hyper-inflammatory condition caused by an uncontrolled, overactive immune status [153]. Viral infections lead to symptomatic disease and complications depending on the underlying vulnerability and the viral load. Thus, vitamin D may not prevent a person from contracting COVID-19 but will reduce symptomatic disease, complications, and deaths. While vitamin D has broader beneficial effects, it is not a cure for everything. For example, in bacterial infections, vitamin D should be used as a supportive therapy to boost the immune system naturally, in addition to primary pharmaceuticals like antibiotics.

## 15. Discussions

A balanced diet with adequate micronutrients, such as vitamins D, B2, K2, and C, and magnesium, trace minerals, and antioxidants, will support a more robust immune system. In most countries, some communities have one or more prevailing micronutrient deficiencies that increase vulnerability to various disorders, such as metabolic, infectious, and non-communicable diseases. In addition to nutrient supplements, fortifying foods with vitamin D and other essential micronutrients will enable them to develop a robust immune system, which prevents them from becoming frequently sick with viral infections.

Nationwide vitamin D supplementation, at least during epidemics and pandemics, markedly reduces acute and chronic diseases, the need for hospitalization, and their complications and deaths, including SARS-CoV-2 infection. It is common to have vitamin D deficiency in those with co-morbidities and chronic diseases, such as hypertension, diabetes mellitus, obesity, and cancer, and to be more vulnerable to developing complications from infections like COVID-19 [154]. Vitamin D sufficiency would reduce the incidence and severity of chronic diseases, such as metabolic disorders (e.g., diabetes, obesity, insulin resistance), cancer, autoimmune disorders, and infections [155,156,157,158]. While the efficacy of vaccines is wading with emerging new mutant Omicron viruses [159,160] and breakthrough SARS-CoV-2 infections [161], the effectiveness of vitamin D will not be affected [55]. Community vitamin D sufficiency is the key to protecting vulnerable populations, especially older people and ethnic minorities with darker skin color, and institutionalized persons [41,122,136,162].

Maintaining serum 25(OH)D concentrations above 40 ng/mL (100 nmol/L) is thought to significantly reduce microbial infections, particularly respiratory viral ones, including COVID-19 [1,163]. Enriching food, such as a targeted food fortification program, is an economical and practical approach for alleviating micronutrient malnutrition in ethnic populations or even for an entire country, as has been carried out with iodine [164]. In the case of COVID-19, those with severe vitamin D deficiency are the most susceptible to complications and deaths, primarily because of weaker immune systems [165]. The addition of other micronutrients, such as zinc and selenium, vitamins A, B2, C, and K_2_, resveratrol, and magnesium, in combination with essential fatty acids, such as omega-3, would facilitate the maintenance of a robust immune system [153,166,167,168] and keep the communities in good health.

Sustained vitamin D deficiency adversely affects human health, which is cost-effectively prevented with vitamin D supplementation and/or regular safe sun exposure. Maintaining population serum 25(OH)D concentrations above 40 ng/mL ensure a robust immune system. Sustained vitamin D deficiency negatively affects all body systems and increases risks for viral infections, outbreaks, and hospitalization. Thus, government and health administrators should consider nationwide educational campaigns for safe sun exposure, vitamin D supplementation, and targeted food fortification programs to strengthen the population’s immunity and keep them healthy. These acts cost less than 0.01% of one day’s hospitalization and significantly reduce healthcare costs.

Health insurance companies have a financial incentive to take proactive actions to keep their clients healthy by maintaining their vitamin D sufficiency. While the efficacy of vaccines and their boosters is wading with emerging Omicron mutant viruses, the effectiveness of vitamin D sufficiency remains solid and unchanged. The key to protecting the vulnerable is maintaining a higher circulatory vitamin D concentration, especially in ethnic minorities, older adults, and institutionalized persons, so they will maintain a robust innate immune system to fight against infections promptly.

Thus, government and health administrations should consider nationwide educational campaigns for safe sun exposure and vitamin D supplemental programs to strengthen the population’s immunity and keep them healthy. Sun exposure and/or vitamin D supplementation and targeted food fortification can achieve this cost-effectively. This would have marked beneficial effects on reducing symptomatic diseases and preventing complications associated with and deaths caused by COVID-19. The world did not capitalize on this highly cost-effective opportunity during the COVID-19 pandemic. This will also protect vulnerable populations (which have a uniformly high prevalence of vitamin D deficiency), such as ethnic minorities with darker skin color, older people, and institutionalized persons.

## 16. Conclusions

Maintaining population serum 25(OH)D concentrations above 40 ng/mL ensures a robust immune system in communities, curtailing the spread of infections, minimizing symptomatic diseases, and reducing the prevalence of chronic diseases. Vitamin D sufficiency also minimizes acute viral infections and outbreaks and the need for hospitalization, saving healthcare costs and lives. Thus, governments, health insurance companies, and health administrators should consider nationwide educational campaigns for safe sun exposure, vitamin D supplementation, and targeted food fortification programs to strengthen the population’s immunity and keep them healthy. Implementing these is less than one day’s cost of healthcare.

While the efficacy of vaccines and their boosters has waded with mutants of Omicron viruses, the effectiveness of vitamin D sufficiency remains strong. Vitamin D has multiple beneficial effects on all body systems: however, it is not a panacea for everything. Apart from preventative use, vitamin D should be used as adjunctive therapy with other primary pharmaceuticals and the best/optimal therapies and approaches, such as using antibiotics in bacterial infections. The key to protecting the vulnerable and reducing chronic disease burden in a country is not by expanding hospitals and health centers and recruiting more healthcare professionals but by educating the public on health preservation and maintaining a higher circulatory vitamin D concentration, especially in vulnerable communities—ethnic minorities, older adults, and institutionalized persons—so that they will have robust immune systems to fight against any infection and minimize chronic diseases.

## Data Availability

Not applicable.
